# Surgical techniques and results of the pulmonary artery reconstruction for patients with central non-small cell lung cancer

**DOI:** 10.1186/1749-8090-8-219

**Published:** 2013-12-01

**Authors:** Qianli Ma, Deruo Liu, Yongqing Guo, Bin Shi, Yanchu Tian, Zhiyi Song, Zhenrong Zhang, Bingsheng Ge, Xiaofei Wang, Thomas A D’Amico

**Affiliations:** 1 Department of General Thoracic Surgery, China-Japan Friendship Hospital, 2 Yinghua East Road, Chaoyang, Beijing 100029, China; 2 Department of Biostatistics & Bioinformatics, Duke University Medical Center, Duke Box 2721, 27710 Durham, NC, USA; 3 General Thoracic Surgery, Duke University Medical Center, Box 3496, Duke South, White Zone, Room 3589, 27710 Durham, NC, USA

**Keywords:** Lung cancer, Pulmonary artery, Reconstruction, Surgery

## Abstract

**Background:**

It is difficult to achieve a margin-negative resection (R0) for non-small cell lung cancer (NSCLC) patients with infiltration of the pulmonary artery. We report our experience of the pulmonary artery reconstruction with regard to long-term survival.

**Methods:**

Clinical records of 118 patients with NSCLC who underwent partial or circumferential pulmonary artery resection during a 21-year period were reviewed retrospectively. Techniques and survival outcomes were analyzed.

**Results:**

We performed 22 pulmonary artery sleeve resections, 51 reconstructions by autologous pericardial patch, 36 tangential resections, 3 left main pulmonary artery (PA) angioplasties during pneumonectomy without cardiopulmonary bypass, and 6 by only preserving the apical and anterior (1^st^) branch of pulmonary arterial trunk. In 41 patients, bronchial sleeve resection was associated; in 7 cases, superior vena cava reconstruction was also required. Thirty-one patients received induction therapy. Thirteen patients had stage IB disease, 41 stage II, 53 IIIA, and 11 IIIB. Ninety-three patients had squamous cell carcinoma, 22 adenocarcinoma, 2 mixed and 1 large cell carcinoma. Negative vascular margins were achieved in all. 5 positive bronchial margins were due to limited lung function. The analysis of 118 cases yielded follow-up data in 94 cases. The mean follow-up was 70 months (range 1–156 months). There was no in hospital death, and the overall 5-year survival was 50.2%. Five-year survivals for stages I and II versus III were 63.9% versus 37.0% (*p* = 0.0059). Multivariate analysis yielded non-squamous cell carcinoma, stage III and patch pulmonary arterioplasty as negative prognosis factors. PA reconstruction associated with bronchial sleeve resection was the positive prognostic factor.

**Conclusions:**

Pulmonary artery resection and reconstruction is feasible and safe, with favorable long-term survival. Our results support this technique as an effective alternative to selected patients with infiltration of the pulmonary artery, such as stage I and II and those who proved down-staged from stage III. Accurate preoperative evaluation, precise and suitable surgical techniques are crucial to achieve good results. Only preserving the anterior and apical pulmonary arteries and reconstruction of the main pulmonary artery by using the artery conduit technique without cardiopulmonary bypass in association with left pneumonectomy can be performed successfully. Postoperative anticoagulation is unnecessary.

## Background

Incidence of non-small cell lung cancer (NSCLC) is continues to increase in many countries, and NSCLC is the leading cause of cancer deaths worldwide [1]. Surgical resection may be curative but complications are often associated. Pneumonectomy has been considered appropriate to achieve cure in patients with the direct invasion of the pulmonary artery (PA) and/or involvement of main bronchus. However, it confers significant higher morbidity and mortality than lobectomy. Pneumonectomy is also associated with reduced quality of life, especially when performed on the right side or after induction chemotherapy [2-4]. These considerations have led to further evaluating a better technique. The origin of PA reconstruction and bronchoplasty surgery can be traced to the end of 1950s. At first, this surgical technique was technically demanding and used only when pulmonary function was compromised to preclude pneumonectomy and its oncologic outcome was in doubt. In the past 2 decades, many thoracic surgeons have confirmed the feasibility and effectiveness of this technique and recommended it should be used when possible. At the present time, reconstruction of PA can achieve complete cancer resection while preserving functioning pulmonary tissue, and has a definite role in the surgical management of lung cancer.

Compared with bronchoplasty, reconstructive procedures of the PA have encountered more difficulties in gaining acceptance. This is due to fewer studies with a large series of patients with long-term follow-up and discouraging results in terms of postoperative complications [5]. We therefore report this retrospective study with introduction of surgical techniques and long-term results after 21 years of legitimate doubts and improving understanding.

## Methods

This retrospective cohort study used an electronic database of consecutive patients between 1990 and 2011, and the study was approved by the China-Japan Friendship hospital Ethics Committee. Patients who underwent partial or circumferential pulmonary artery resection and had margin-negative resections at China-Japan Friendship hospital were eligible. Different non-small cell lung cancer TNM stage editions existed during the last 21 years. The data was collected with pathology reports, so we reviewed the pathological details and use only one standard. That is 7^th^ edition, 2009.

### Evaluation

Entry criteria for this study mandated clinical history, physical examination, computed tomography (CT) scan with intravenous contrast no more than 1 month before resection, pulmonary function test, blood gas analysis, cardiac evaluation, bronchoscopy, abdominal B-ultrasound, cerebral magnetic resonance imaging (MRI), isotopic bone scanning and basic examinations as usual. Fluorodeoxy-glucose-position emission tomographies were performed in selected cases. Patients who had biopsy-proven N2 disease received neoadjuvant chemotherapy with a cisplatin-based regimen. Preoperative radiotherapy is not routinely used at our center. All patients were restaged after neoadjuvant therapy, and resection was generally reserved for those who were down-staged. Surgical technique for PA and bronchial reconstruction was uniform throughout the study period. Operative mortality was defined as any death within 30 days of surgery or during the same hospital admission.

### Surgical technique for pulmonary artery resection and reconstruction

**Figure 1:**
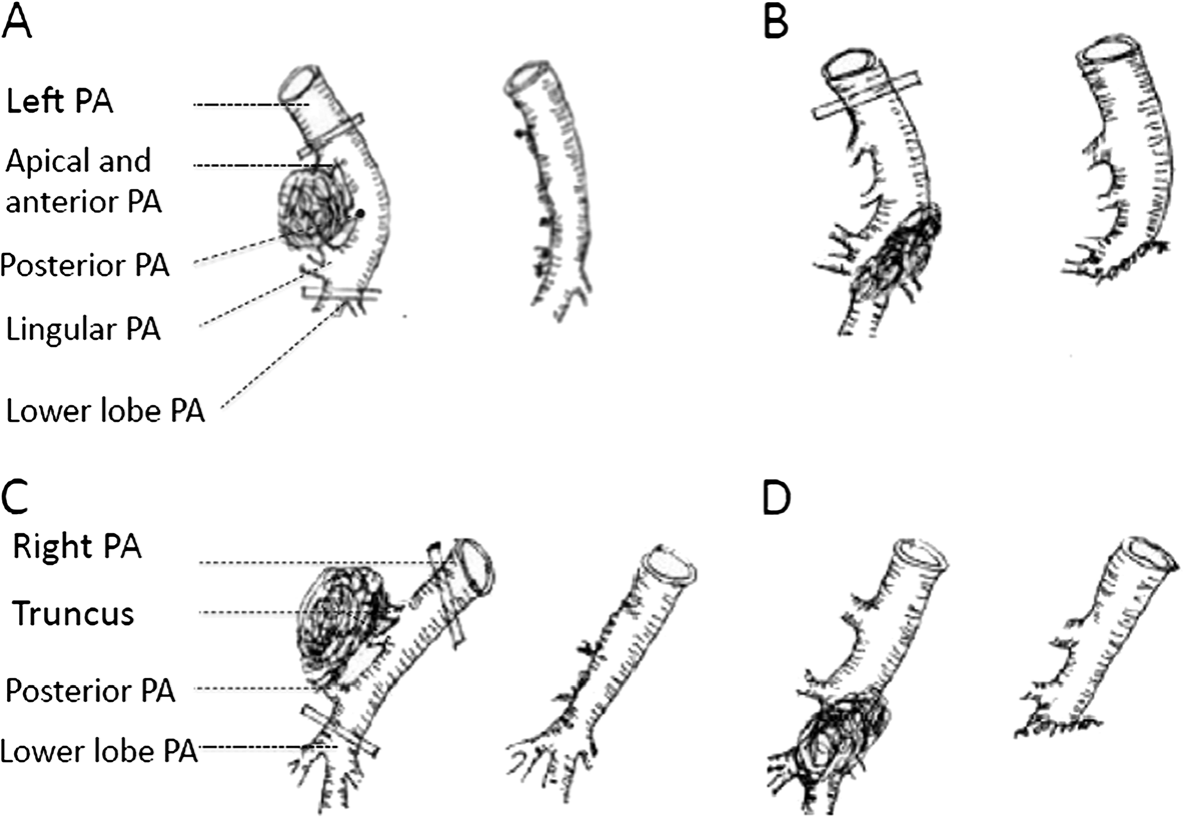
**Tangential arterial resection with direct sutures. A**. Left upper lobectomy. Left apical, anterior and lingular arteries were invaded. **B**. Left lower lobectomy. Left common basal and superior segmental arteries were invaded. **C**. Right upper lobectomy. Right truncus anterior and posterior ascending arteries were invaded. **D**. Right lower lobectomy. Right common basal and superior segmental arteries were invaded.

**Figure 2:**
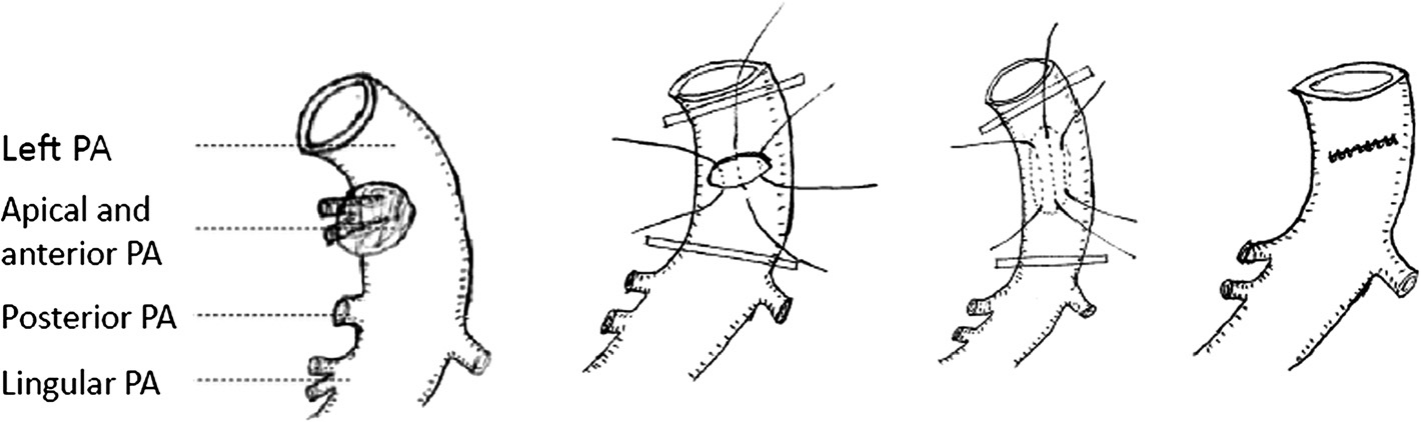
**The left main pulmonary artery wall was resected along the vertical axis of main PA (around the invasion) and then primary repair was performed along the horizontal axis.** Left anterior and apical pulmonary arteries were invaded.

Once proximal and distal control of the PA was obtained, infiltration area of PA wall was removed sharply. The artery was reconstructed to reestablish blood flow to the remaining pulmonary parenchyma. Suturing was completed with double continuous 5–0 polypropylene sutures. Tangential arterial resection with direct sutures was carried out when possible (residual arterial caliber, >70%), as shown in Figure 1. Sometimes the length of the invasion was long but the width was narrow. To avoid a significant stenosis, the artery wall was resected along the vertical axis of PA (around the invasion) and then primary repair was performed along the horizontal axis, as shown in Figure 2.

**Figure 3:**
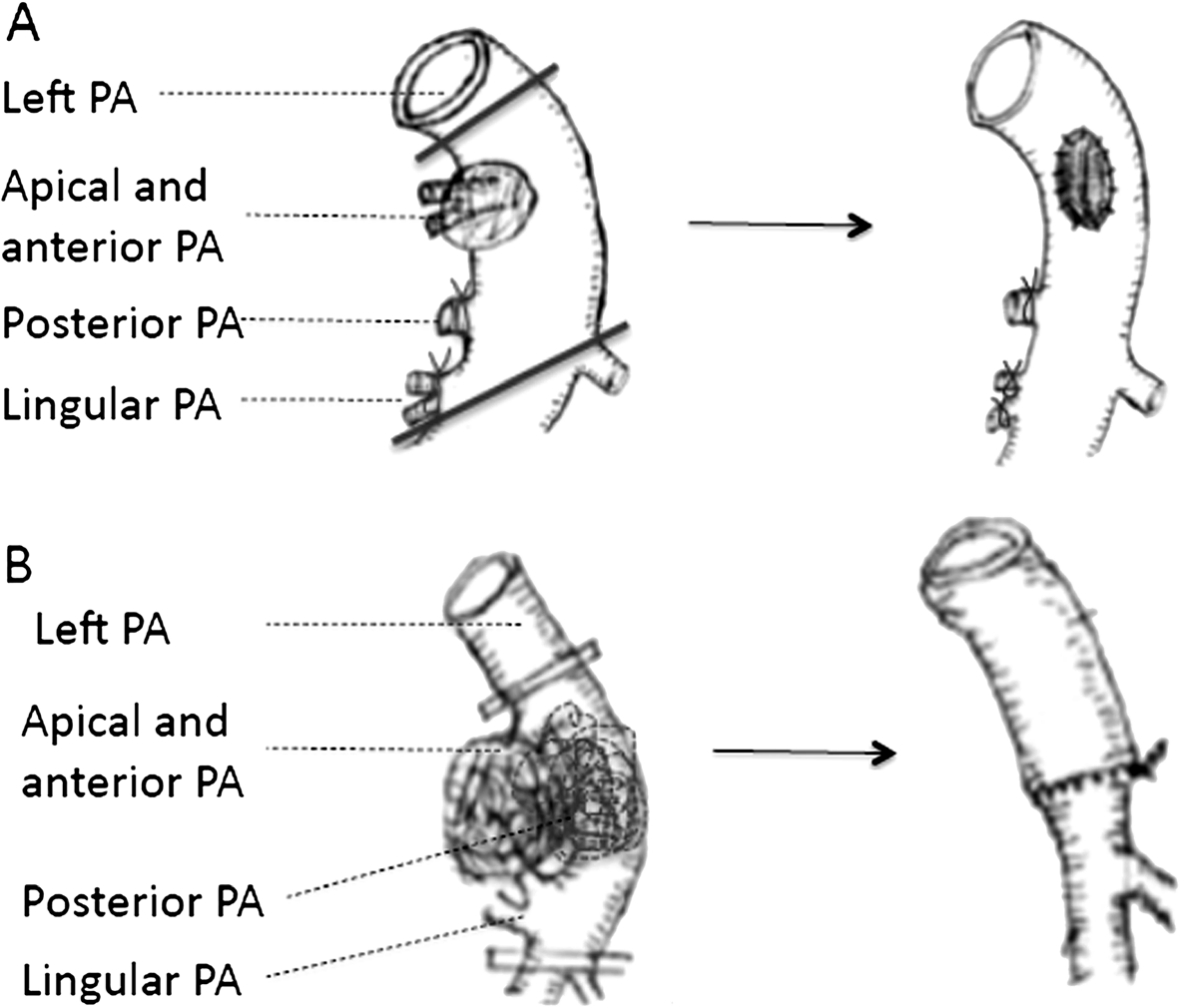
**Patch and circumferential resection. A**. Left apical and anterior arteries were invaded. **B**. Left apical, anterior, and lingular pulmonary arteries were all invaded.

Otherwise, application of autologous pericardial patch (PA invasion was from 30% to 50%) or circumferential resection (PA invasion was more than 50%) was performed. The vessel should be carefully preserved with a sufficient lumen. And the distal occlusive clamp was released before suture tie to remove air, as shown in Figure 3.

**Figure 4:**
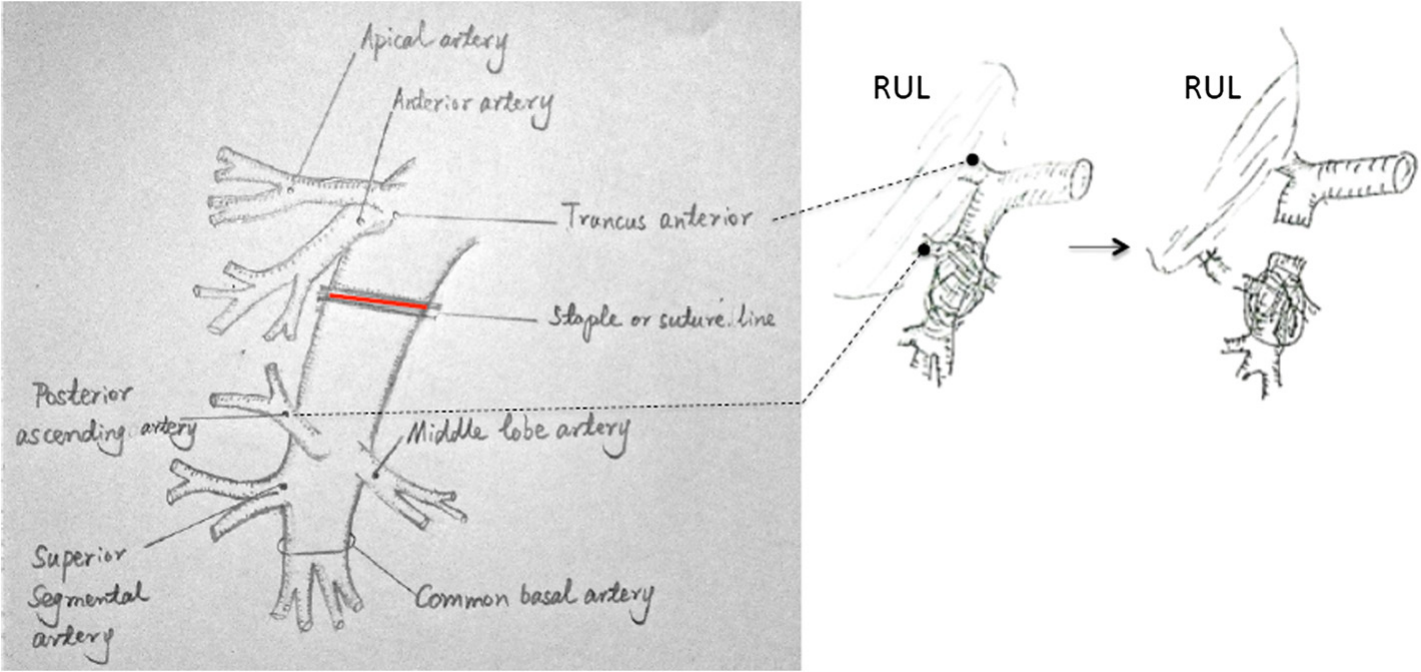
Only preserved the truncus anterior (both anterior and apical pulmonary artery branches) for bilobectomy (Right middle & lower lobectomy).

**Figure 5:**
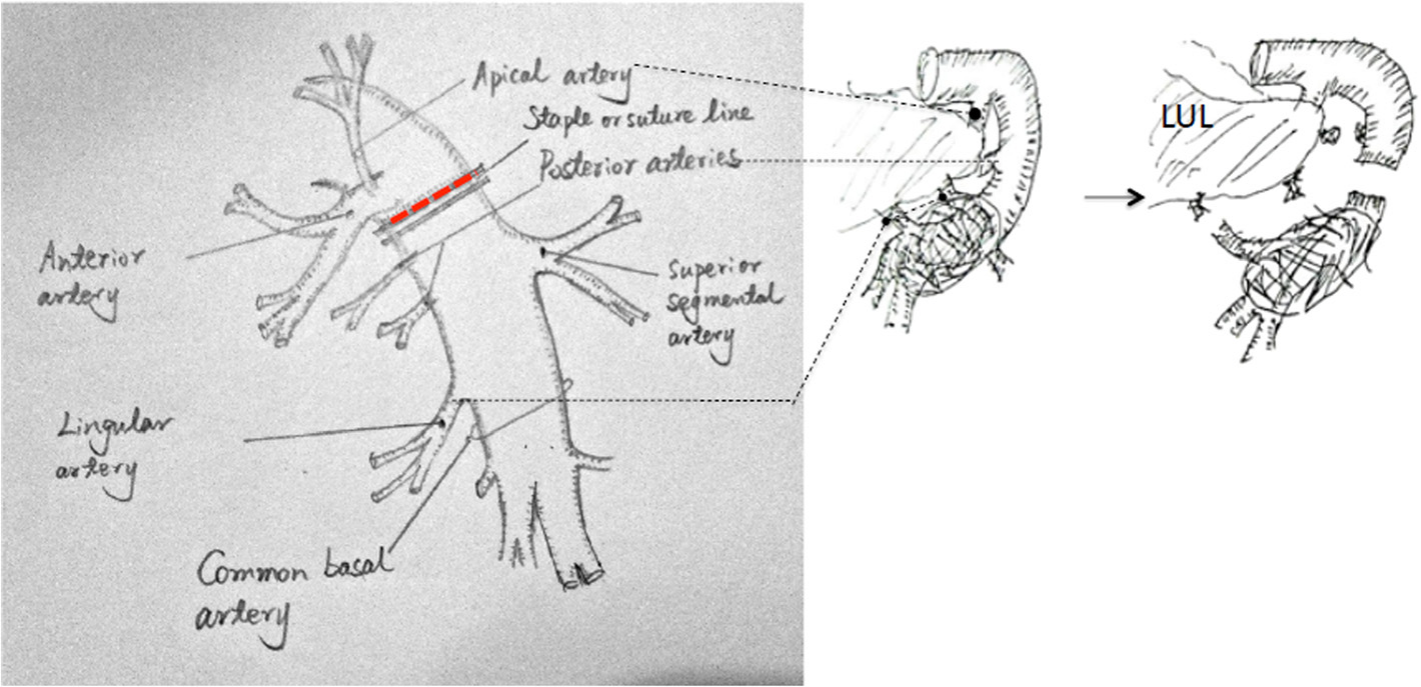
Only preserved the anterior and apical pulmonary artery branches for Left lower lobectomy.

In a few cases, the pulmonary ateries were intensively infiltrated below the level of the 1^st^ branch (anterior and apical pulmonary arteries) by tumor or lymph nodes. We only kept the 1^st^ branch to perfuse the entire right or left upper lobe, and then performed bilobectomy (right middle & lower lobes) or left lower lobectomy, as shown in Figure 4 and Figure 5. If the space for surgery was limited and the 1^st^ branch originated more proximal to heart, we suggest opening pericardial sac to expose the root of main pulmonary artery. Next, an occlusive clamp at the root level of main PA can be placed to facilitate safer control of the inflow. There were 6 patients who received this surgery, and no evidence of insufficiency of blood infusion or hypoxemia was found. Postoperative outcomes were acceptable.

**Figure 6:**
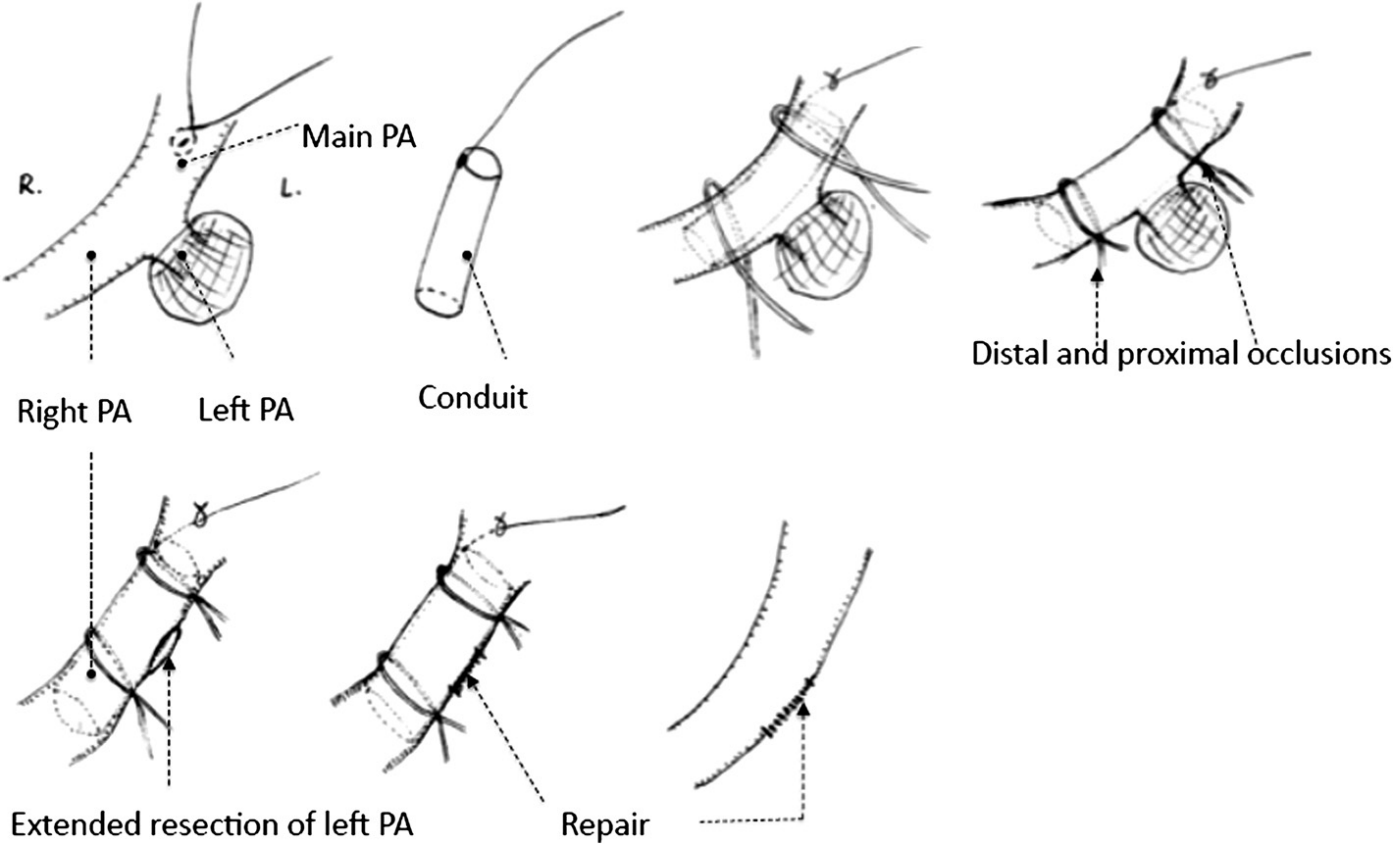
**Temporary intra pulmonary artery conduit technique was used for 3 patients who underwent left pneumonectomy.** The root of the left pulmonary artery (bifurcation of the main pulmonary artery) was invaded.

The invasion of the left main PA root occurred quite infrequently. Cardiopulmonary bypass (CPB) was always used to control of the main pulmonary’s blood flow. Another option, the temporary intra-artery conduit technique, was recommended here. This procedure was used for 3 patients who underwent left pneumonectomy. Once the conduit was inserted from the main PA to the right PA, proximal occlusion of the main PA and distal occlusion of the right PA were then obtained. Repair of the defect could be performed after extended resection of left main PA. Distal occlusion was released first to remove air and test if the repair was satisfied. And then the continuity of the main PA could be regained immediately after conduit was removed from the arterial lumen. In these 3 cases, control of the main PA blood flow could be achieved by using this technique without CPB. This procedure can be performed with less operative cost, fewer complications, decreased length of stay and more technical ease. They were proved had no hemodynamic consequences by trans sternal echocardiogram, as shown in Figure 6.

We stapled the pulmonary vein first and then injected 1500U of heparin intravenously. Next, two Satinsky clamps were placed on the proximal and distal PA. The handles of clamps were positioned facing in the opposite direction. This position afforded more space for the surgeons. The width of the arterial wall is always limited for angioplasty. Therefore, frozen section of the arterial wall is necessary to ensure the negative margin. In order to decrease the PA occlusive time, Rendina and colleagues reestablished the PA continuity first, when circumferential resections of the bronchus and artery were both required [6]. However, we thought this would increase tension of the sewn PA or PA anastomosis. Thus we performed the bronchus anastomosis with interrupted sutures first, followed by the PA resection or anastomosis. The bronchial anastomosis was covered with an intercostal muscle flap. An autologous pericardial patch was used when the residual PA lumen was narrowed from 30% to 50% of its original size after partial resection of the arterial wall. When the invasion is more than 50%, we preferred circumferential resection and sleeve anastomosis, protected with mediastinal pleura.

### Follow -up

Follow-up information was obtained from several sources, including telephone interviews with patients or their family, Social Security Death Index, clinical letters or letters from oncologists and other physicians. There were 24 patients unavailable for follow-up at the completion of study. The mean follow-up was 70 months (range 1–156 months). Patients were followed postoperatively with chest CT and blood tumor markers every 6 months in the first 2 years, and yearly there afterwards. We agree that the use of these markers is not standard in North America, South America or Europe, but the markers are commonly used in Asia. Fludeoxyglucose Positron emission tomography (FDG-PET) was utilized in patients with a suspicion of metastatic disease or mediastinal involvement: the shortest diameter of the enlarged mediastinal lymph node was more than 1 centimeter on the chest CT. Due to financial constraints, PET could not be utilized in all patients.

### Statistical analysis

Data were collected and stored with an Excel database (Microsoft Corp, Redmond, Wash). Overall survival rates were calculated by using the Kaplan-Meier method and compared with the Log-rank test. All of the clinical and pathologic variables with a possible effect on survival were entered in a multivariate analysis (Cox proportional hazard model) to identify independent prognostic factors. The statistical analysis was performed with the SAS software, version 9.3 (SAS Institute, Cary, NC).

## Results

Between April 1990 and April 2011, 3095 Lung resections were performed in our department: 364 pneumonectomies, 2150 lobectomies and 220 sleeve lobectomies. The population of this study was 118 pulmonary artery reconstructions including 18 dual (broncho-vascular) sleeve lobectomies, 97 lobectomies and 3 pneumonectomies.

### Preoperative data

There were 87 men (75.7%) and 28 women (24.3%). The mean age was 61 ± 12.2 years. The mean preoperative FEV1 was 78.5% ± 18%. Percentage of smoker was 80.5%. 37 patients had comorbidities (hypertension n = 21; arrhythmia n = 3; previous myocardial infarction n = 2; chronic obstructive pulmonary disease n = 5; carotid artery atherosclerotic disease n = 1; cerebrovascular disease n = 2; diabetes n = 8). Induction chemotherapy was carried out in 31 patients (28 received chemo therapy alone and 3 received chemo radiation therapy). All chemotherapy protocols were platinum based and the main indication for neoadjuvant therapy was N2 disease.

### Surgical and pathologic data

Thirty-seven resections were performed on the right side and 81 on the left (3 left pneumonectomies were included). Partial PA resections with primary closure were carried out in 36 (31.3%) patients. Patch pulmonary arterioplasties were performed in 51 (44.3%). Patches were made with autologous pericardium. Twenty-two patients underwent circumferential resection and end-to-end anastomosis. The apical and anterior pulmonary artery branches preserved procedures were accomplished in 6 patients. Bronchial sleeve resection was associated in 41 patients (intercostal muscle was harvested and wrapped around the bronchial anastomosis). In 7 cases, superior vena cava reconstructions were also required.

Thirteen patients had stage IB disease, 41were in stage II, 53 were in IIIA and 11 were in IIIB. Two groups of stage I and II were combined for survival analysis due to relatively small numbers. Histological examination showed that ninety-three patients had squamous cell carcinoma, 22 adenocarcinoma, 2 mixed and 1 large cell carcinoma. The proportion of squamous cell carcinoma (79.1%) is significantly higher than other types.

### Postoperative outcome

Although the overall morbidity was 52.2% (n = 60), and there were no intraoperative deaths. The most frequent complication, supraventricular arrhythmia, occurred in 29 cases (25.2%). One pulmonary artery thrombosis was demonstrated by angiography after a left upper sleeve lobectomy with PA end-to-end reconstruction. This patient underwent successful pneumonectomy (on postoperative day 3). Two patients with respiratory failure required reintubation on postoperative days 2 and 4, respectively. Other complications were pulmonary edema (n = 4), atelectasis (n = 5), pneumonia (n = 8), left recurrent laryngeal neurapraxia (n = 2) and prolonged air leak (n = 11). Long-term postoperative anticoagulation is not required if no other disease coexist. Self-compressive leg device is used to help prevent deep venous thrombosis and pulmonary embolism. Patients undergoing the temporary intra-artery conduit technique died because of distant metastasis. These 3 patients were excluded from the survival analysis.

### Long-term outcomes

**Figure 7:**
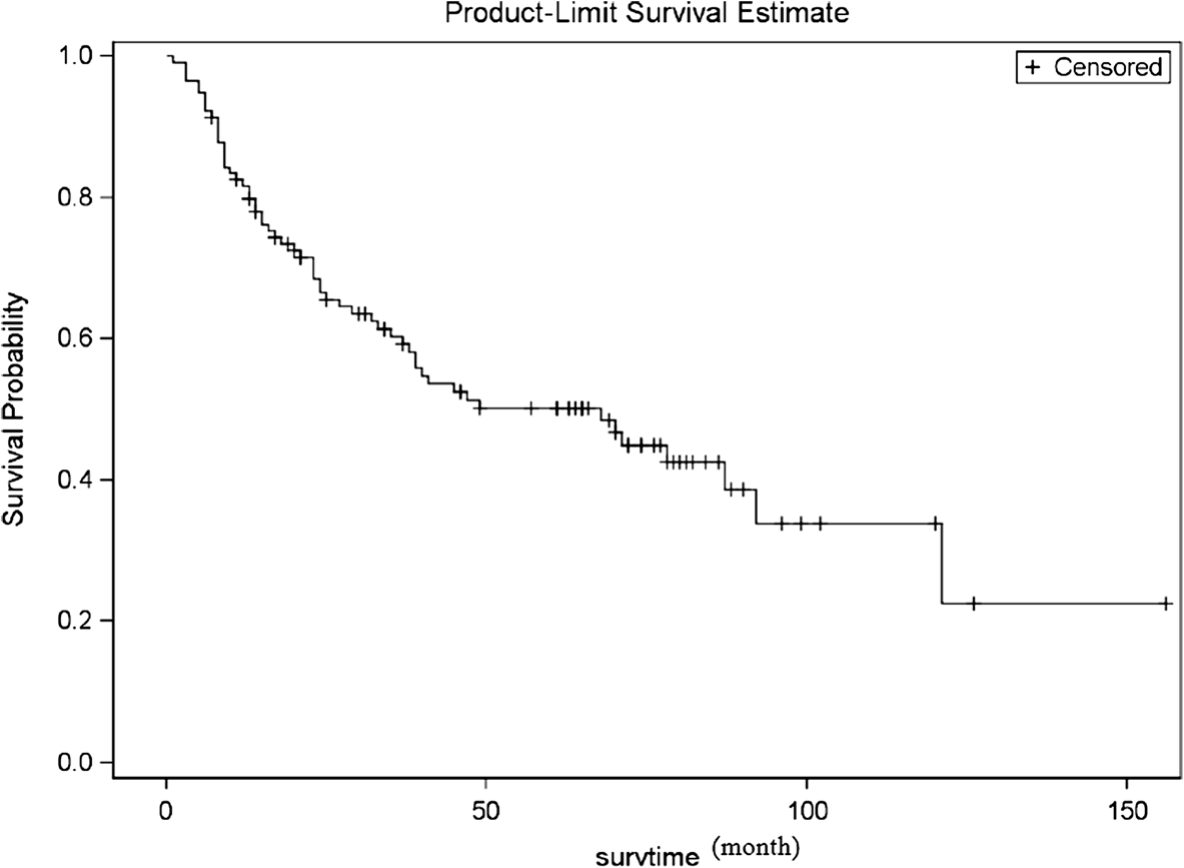
**Overall survival curve.** 5-year survival rate is 50.2% (95%CI: 40.0%-59.5%, calculate on 115 cases).

For the whole population (calculated on 115 patients, 3 left pneumonectomy were not included), the median survival was 68 months (95%CI: 63–77 months); overall 3- and 5-year survival was 60.3% (95%CI: 50.1%-68.9%) and 50.2% (95%CI: 40.0%-59.5%), respectively (Figure 7).

113 (95.8%) patients received R0 resection. The 5 cases with positive bronchial margins were due to limited lung function. They were deemed not to tolerate sleeve pneumonectomy. Regarding the recurrence rate (47.8%), three factors may be responsible. First, 35 patients were confirmed to have with ipsilateral mediastinal lymph nodes metastasis (N2). Of these 35, 24 were found between 1990–2000. PET-CT, mediastinoscopy, and EBUS (Endobronchial Ultrasound-guided Trans Bronchial Needle Aspiration) were regularly employed after that. Second, five patients developed secondary tracheal tumors after sleeve lobectomy. There were no sleeve margins microscopically positive on final pathology. These five secondary tumors are separate airway tumors, and have identical histology compare to the original tumor. Third, the remaining patients with recurrence had both local and distant disease.

The incidence of complications, nodal involvement, and gender were found to be independent perioperative variables related to recurrence after complete surgical resection. Odds Ratio estimates are 378.2 (95%CI: 36.5-1000), 28.2 (95%CI: 2.9-276.2) and 6.6 (95%CI: 1.0-43.7), respectively. Locoal regional recurrence is defined as any recurrence that occurred with in the ipsilateral hemithorax including mediastinum or neck area. These could be isolated or part of wide spread (local and distant) recurrent disease. Six isolate pulmonary nodules of NSCLC were treated with wedge resection; 7 tracheal recurrences were managed with endo-tracheal tumor cryotherapy. Two of them occurred by virtue of positive margins. Five cases were secondary tumors. Eighteen multiple pulmonary nodules (10 distant metastasis included) and 21 extra-thoracic recurrences were managed with chemotherapy, 5 thoracic radiotherapy and 3 brain radiotherapy were associated. A patient with isolated adrenal metastasis, one patient with anterior chest wall recurrence and two isolated brain metastasis underwent surgical intervention. Among the 59 late deaths, 4 died from non-tumor associated causes: 1 myocardial infarction, 1 car accident, 1 stroke, 1 renal failure and pneumonia. The remaining was due to recurrent disease.

**Figure 8:**
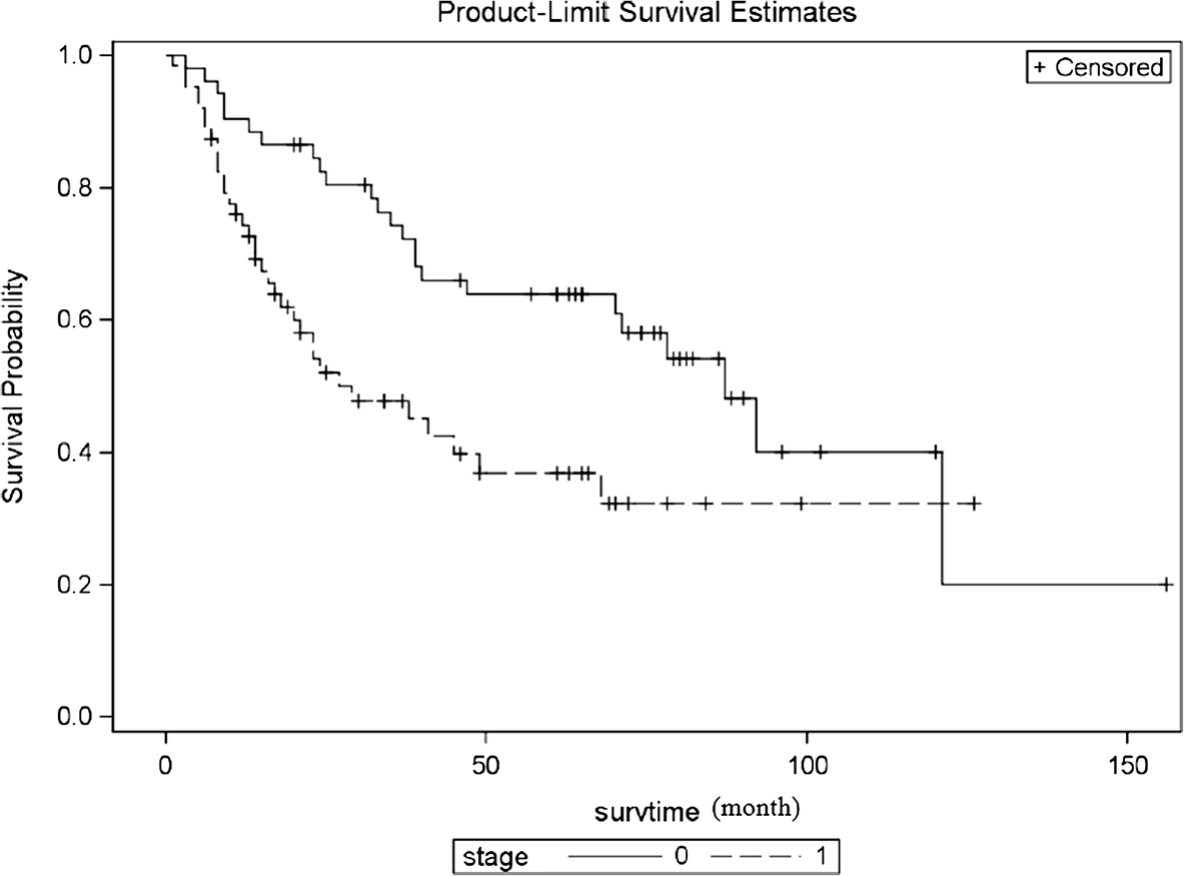
**Survival curves by stage (0 = stage I and II, 1 = stage III).** Five-year survival for stages I and II, versus III were 63.9% versus 37.0% (*p* = 0.0059), calculate on 115 cases.

Patients with stage I and II had a significantly better 5-year survival than stage III (63.9% vs. 37.0%, *p* = 0.0059), as shown in the Figure 8. No significant differences were found with respect to age, gender, comorbidities, choice of reconstruction techniques, and possible associated bronchoplasty. A trend toward better outcome was observed in patients with squamous cell carcinoma compared with non-squamous cell carcinoma (5-year survival of 53.8% vs. 37.8%, *p* = 0.080). Patients without induction therapy showed a trend toward better outcome compared with have neoadjuvant therapy (54.8% vs. 38.3%, *p* = 0.085). Patients with N0 or N1 disease had a better outcome than N2 disease (55.4% vs. 37.8%, *p* = 0.093), as shown in Table 1.

**Table 1:** Univariate analysis for 5-year survival

**Variable**	**NO**	**%**	**5-year survival (%)**	**95% CI (%)**		***P *** **value**
				**SDF_LCL**	**SDF_UCL**	
Gender						0.4340
Male	87	75.7	50.3	38.5	61.1	
Female	28	24.3	50.6	30.0	67.4	
Age						0.2477
≥65y	64	55.7	45.7	31.9	58.4	
<65y	51	44.3	55.8	40.5	68.7	
Stage						0.0059
I and II	52	45.2	63.9	48.9	75.6	
III	63	54.8	37.0	23.4	50.6	
Comorbidity						0.2309
Yes	37	32.2	62.7	44.3	76.5	
No	78	67.8	44.6	32.5	56.6	
Induction						0.0854
Yes	31	27.0	38.3	20.1	56.3	
No	84	73.0	54.8	42.9	65.2	
Histologic type						0.0802
Squamous	91	79.1	53.8	42.3	64.0	
Non-squamous	24	20.9	37.8	18.4	57.1	
Nodal status						0.0933
N0 ~ N1	80	69.6	55.4	43.2	66.0	
N2	35	30.4	37.8	20.1	55.4	
Reconstruction tech.						0.1723
Tangential R.	36	31.3	57.6	37.8	73.1	
Patch	51	44.3	40.0	26.0	53.6	
Sleeve	22	19.1	55.3	31.2	74.0	
Truncus preserved	6	5.2	83.3	27.3	97.5	
Procedure						0.1462
PA alone	74	64.3	43.1	30.8	54.9	
PA + B. sleeve	41	35.6	63.8	46.5	76.8	

PA alone = patients only received pulmonary artery reconstruction.

PA + B. sleeve = patients received pulmonary artery reconstruction associated with bronchial sleeve resection.

Calculated on 115 patients, those who underwent left pneumonectomy with artery conduit technique (n = 3) were not included.

The PA resection and reconstruction procedure associated with bronchial sleeve resection was a independent factor of good outcome. It is a protective factor (Hazard Ratio = 0.203, *p* < 0.0001), during multivariate analysis. While, histology type of non-squamous cell carcinoma, stage III and patch pulmonary arterioplasty were independent factors of worse outcome (*p* = 0.0007, 0.0012 and 0.0208 respectively), as shown in Table 2.

**Table 2:** Multivariate analysis for 5-year survival

**Variable**		**Hazard ratio**	***P *** **value**
Gender	Male vs. female	1.781	0.0978
Stage	III vs. I&II	2.819	0.0012
Histologic type	Non-squamous cell carcinoma Vs. squamous cell carcinoma	4.536	0.0007
Procedure	PA reconstruction associated with bronchial sleeve resection vs. not	0.203	<0.0001
Reconstruction technique of PA	Patch vs. tangential	2.138	0.0208
	Sleeve vs. tangential	0.798	0.6357
	Truncus preserved vs. tangential	0.175	0.1200

## Discussion

Reconstruction of the pulmonary artery usually indicates that tumors or lymph nodes adhere to or invade the PA in the hilum and preclude complete resection by simple lobectomy. The PA can be compromised to various degrees, from partial filtration to a more extensive and even circumferential invasion or grow inside of the lumen. This heterogeneous presentation require different types of techniques, like tangential resection with direct repair, patch reconstruction, end-to-end anastomosis or interposition of a prosthetic conduit [6].

Accurate evaluation of the PA involvement has been improved with the help of three-dimensional reconstructions imaging. However, presence of vascular encroachment cannot be defined with interpreting imaging and it is impossible to make a specific PA reconstructive plan preoperatively. The most appropriate technique option of reconstruction is usually chosen during the operation on the basis of how much the vascular is invaded, and this crucial decision may take a long time and many efforts. Once face this situation surgeons should be encouraged to avoid the easier choice, pneumonectomy.

In our experience and in agreement with others [7,8], it is much more frequently to perform PA reconstruction on the left side and for squamous cell carcinoma (SCC). SCC is more often to be seen in central type NSCLC because larger bronchi are usually the primary sites of this type tumor. And it often metastasizes to hilar lymph nodes early in its course. Compared with the right side, both superior and posterior side of left upper bronchus is surrounded by the PA. Due to this anatomical reason, direct invasion by primary tumor or metastasis lymph nodes originating in the lung hilum is more likely to occur on the left side. Similar to lobotomies, the 3 pneumonectomies were also on the left side. In addition, the left recurrent laryngeal nerve is also easier to be injured compared with right side, care should be taken when using energy equipment and extra extend retraction should be avoid.

Induction chemotherapy is usually used for patients with N2 or chest wall invasion [9], and it is usually more technically demanding after induction therapy, because of the deleterious effects on bronchial healing, vascular fragility and local devasculation. Safety can be guaranteed once the proximal PA is clamped. If there was no enough room to place the clamp, open the cardiac sac to expose the PA more clearly could be an option. When bronchial sleeve resection is needed, we prefer to protect the bronchial anastomosis by intercostal muscle flap. It can act as a buffer between PA and bronchus, and this buffer should not be too thick. That would elevate pulmonary artery too high and reduce the blood inflow.

We found that tumor or metastatic lymph nodes located in the lower or middle lobe occasionally just invaded the upper lobe along the interlober PA. The upper lobe could be preserved as long as a clean arterial margin could be achieved below the level of the 1st branch (apical and anterior branches) of the pulmonary artery. Complications occurred in 3 out of 6 (50%). They are empyema (n = 1 because of his nephron syndrome), pneumonia (n = 1), and transient atria fibrillation (n = 1). 2 lived more than 5 years, 1 died of myocardial infarction 2 years later, one died of brain metastasis 3 years later and 1 died of respiratory failure 18 months later. One was lost during follow-up. No other authors have reported this kind of technique before. In our limited experience of these 6 patients, the upper lobe can be preserved with this viable option. The blood supply for the upper lob is sufficient just relying on the 1^st^ branch of pulmonary artery. Patients had better postoperative pulmonary function and improved quality of life. However, more cases are needed when analyzing the long-term outcome. The temporary intra-artery conduit technique brought us some conveniences and the patients can recover faster with lower cost. Nevertheless, the potential higher risks in thesis cases are not insubstantial. We only utilize this technique when the circumferential encroachment of the main PA is less than 20%. And CPB should be prepared before and during the operation.

The incidence of complications in our study was 52.2% and was associated with older age (*p* < 0.0001), Stage III disease (*p* < 0.0001), induction chemotherapy (*p* < 0.0001), non-squamous cell carcinoma (*p* = 0.0006), N2 status (*p* < 0.0001) and PA extended resection with bronchus sleeve resection (*p* < 0.0001). Concern about high postoperative complications limited the acceptance of the PA angioplasty [10]. In fact, they are companied with the operation and most of them can be controlled with medication successfully without influence of the 5-year survival. How the incidence of complications significantly predict recurrence was uncertain to the authors. It is possible that the development of complications is a surrogate for patients with more extensive disease (not captured by the staging system), for example, more proximal tumors or magnitude of N1 adenopathy. In these patients, the resection is more difficult, which may lead to a higher complication rate, and the recurrence rate (local and regional) might also be higher. This finding only suggests an association between these two events—not that patients should not have undergone resection—but whether surgical complication caused recurrence is another question to be tested in future studies.

The estimated 5-year survival rate was 50.2%, which is consistent with that reported for standard resection [11]. The results from the present study suggest that stage III,histology type of non-squamous cell carcinoma and patch pulmonary arterioplasty are prominently prognostically related. The 5-year survival for stage III is significantly lower than stage I and II. Although it is more technical demanding after chemotherapy and/or radiation therapy, reconstruction of PA can be carried out safely. So we recommend induction and/or radiation therapy first for the patients with stage III, and those who are proved down-staged would be selected for surgical candidates. Those who progress through therapy are clearly not going to benefit from resection and should be considered inoperable. Decision of operation for patients who had no change after induction is complex. The benefits of resection and benefits of non-operative therapy should be carefully deliberated in multi-disciplinary tumor board and informed to the patents.

Non-squamous cell carcinoma showed more aggressive biological character and generally disseminates outside the thorax somewhat earlier than squamous cell carcinoma. These patients should be considerate as a different group and treated separately. Tineke W.H. Meijera and his colleagues found this was due to the difference in metabolism. Adenocarcinomas may use aerobic glycolysis as an energy source, whereas the metabolism of squamous cell carcinomas seems to rely on mitochondrial oxidation with anaerobic glycolysis under hypoxic conditions. Aerobic glycolytic metabolism is an additive responsible factor for aggressive behavior in NSCLC [11]. This subgroup of tumors may try new treatment approaches first, such as MCT4 inhibitors, and then consider surgery.

When considering appropriate surgical technique, careful examination and precise evaluation of the extent of PA involvement is critical. Errors should be avoided when choosing reconstruction techniques. The goal of the angioplasty was to obtain a suitable length of PA and smooth lumen. Tension and bleeding were probably resulted from inadequate artery length. On the other hand, the axis of PA was more easily twisted due to unnecessary preservation. The vessel twist might impair the blood flow and lead to the stenosis or thrombosis. When upper lobectomy was performed, more attention should be paid to avoid the kinking because of elevation of the lower lobe. Autologous pericardial patch can exceed the length of PA, and there is no posterior suture line on the artery, which can be a buffer against the bronchial stump or anastomosis. However, it is difficult to harvest patches with suitable size. Because it was hard to estimate how much the graft will stretch, shrink kinking or turbulence after the artery was reexpanded [12].

We recommend aspirin (100 mg per day) for high-risk patients 24 hours after PA plasty. This 24 hours’ time window was used to observe the volume and character of chest drainage to exclude post-operative hemothorax. High-risk patients included hypertension, coronary atherosclerosis, and more than 50 years old. Contraindications are the volume of chest bloody drainage per day is more than 200 ml, peptic ulcer, trend of stoke and allergy to aspirin. In this study, stents or grafts were not used, so we did not have experience of other anti-platelet like clopidogrel. For the issue of preventing pulmonary embolism, Heparin lock flush solution (12500 u diluted with 500 ml sodium chloride solution) was used during operation. Considering the pulmonary artery blood flow was broken off when proximal and distal parts of the PA were both controlled, intravenous heparin sodium (1 mg/kg) was used when applying autologous pericardial patch or circumferential resection.

Complete resection and long-term survival are mainly affected by neoplastic events. PA reconstruction associated with bronchial sleeve resection is a positive prognostic factor in our study. This can be expained by more complete resection percentage and lower risk of recurrence. The study also shows that PA invasions are more longitudinal and circumferential for the patients who received patch angioplasty. Their 5-year survivals are significantly lower than others.

## Conclusion

The retrospective study method, different preoperative status and heterogeneous of stage, nodal and histology are limitations of this study. We cannot draw some confirmative conclusions. Nevertheless, we do not found local recurrence at the level of the PA angioplasty or other oncological or technical drawbacks. The early and long-term results suggest that these surgical techniques of PA reconstruction are feasible and safe, and the postoperative morbidity is also acceptable. Stage I and II and those who are proved down-staged from stage III should be selected for surgical candidates. It is reasonable to apply this technique more liberally as long as R0 resection is achieved, regardless of pulmonary function.

## Abbreviations

NSCLC: Non-small cell lung cancer; PA: Pulmonary artery; R0: Margin-negative resection; CT: Computed tomography; CPB: Cardiopulmonary bypass; FDG-PET: Fludeoxyglucose positron emission tomography; SCC: Squamous cell carcinoma; TNM: Tumor node metastasis; EBUS-TBNA: Endobronchial ultrasound-guided trans bronchial needle aspiration.

## Competing interest

The authors declare that they have no competing interests.

## Authors’ contributions

XM participated in the collection of the data. YP participated and performed the statistical analysis. CL conceived of the study. HZ participated coordination. All authors read and approved the final manuscript.

## Authors’ information

QM graduated from Tongji Medical University, Wuhan, Hubei, China on 06/30/2006, and received the Qualification Certificate of Specialty and Technology on 05/10/2009. (Approved & authorized by Ministry of Human Resources and Security and Ministry of Health, The People’s Republic of China). His current position is fellow in the department of General Thoracic Surgery, China-Japan Friendship Hospital. QM is the member of Chinese Society for Thoracic and Cardiovascular Surgery (CSTCVS), and Chinese Association of Thoracic Surgeons (CATS).
